# Recent advances in the diagnosis and management of sepsis in pregnancy

**DOI:** 10.12688/f1000research.18736.1

**Published:** 2019-08-30

**Authors:** Amaan Ali, Ronnie F Lamont

**Affiliations:** 1 St Bartholomew’s and The London School of Medicine and Dentistry, 4 Newark St, Whitechapel, London, E1 2AT, UK; 2 Department of Gynecology and Obstetrics, University of Southern Denmark, Institute of Clinical Research, Research Unit of Gynaecology and Obstetrics, Kløvervænget 10, 5000 Odense C, Denmark; 3 Division of Surgery, University College London, Northwick Park Institute for Medical Research Campus, Watford Road, London, HA1 3UJ, UK

**Keywords:** Diagnosis, Infection, Management, Pregnancy, Sepsis, Treatment.

## Abstract

**Background:** Maternal sepsis accounts for 11% of all maternal deaths worldwide. It is the third most common direct cause of maternal death and is a major contributor to other common causes of maternal death, such as haemorrhage and thromboembolism. **Methods: **This review addresses important topics, including the epidemiology, risk factors, prevention, diagnosis, care bundles and management of maternal sepsis, including antibiotic treatment, and critical care interventions such as extracorporeal membrane oxygenation. Preventative measures that have had an impact on maternal sepsis as well as future research directions are also covered in this review. Case studies of maternal sepsis which highlight key learning points relevant to all clinicians involved in the management of obstetric patients will also be presented. **Results:** Although, historically, maternal death from sepsis was considered to be a problem for low-income countries, severe obstetric morbidity and maternal death from sepsis are increasing in high-income countries. The global burden of maternal sepsis and the obstetric-related and patient-related risk factors and the likely sources are presented. Recent changes in definition and nomenclature are outlined, and challenges in diagnosis and identification are discussed. **Conclusions** **:** Following maternal sepsis, early diagnosis and early intervention are critical to save lives and prevent long-term adverse sequelae. Dogma surrounding critical care interventions in pregnancy is being challenged, and future research is warranted to maximise therapeutic options available for maternal septic shock.

## Introduction

Maternal sepsis accounts for 11% of maternal deaths worldwide and is the third most common direct cause of maternal death ^1^. In addition, sepsis contributes to other common causes of maternal death, such as haemorrhage and thromboembolism. Despite the increased mortality and morbidity in pregnancy and the unpredictable nature of emerging causative organisms (such as novel influenza serotypes), maternal sepsis has not attracted the same attention and research as other leading causes of maternal death ^2^. Failure to recognise sepsis early is a significant cause of preventable morbidity, resulting in delayed treatment and escalated care, which are critical if lives are to be saved ^3^. Our understanding of the pathophysiology of sepsis has markedly improved, and there is a greater appreciation of the interplay between maternal physiology and sepsis, which has important implications for the diagnosis of sepsis during the antenatal, intrapartum and postpartum periods ^4^.

## The global burden of maternal sepsis

While sepsis is estimated to cause 9.7%, 11.6% and 7.7% of maternal deaths in Africa, Asia and Latin America/Caribbean respectively, maternal death from sepsis is also increasing in high-income countries ^5– 9^. The greatest burden of sepsis-related deaths occurs in the region of Southern Asia, where sepsis accounts for 13.7% of all maternal deaths ^1^.

The US Nationwide Inpatient Sample demonstrated an annual 10% rise in maternal deaths between 1998 and 2008 ^10^. Investigators have suggested various factors, including increased antibiotic resistance, maternal age, co-morbidities and microbiological factors such as an increased incidence of *Escherichia coli* and group A streptococcal infections ^7^. In 2014, the UK Obstetric Surveillance System reported a prospective case-control study of 365 confirmed cases of severe maternal sepsis and 757 controls from all UK obstetrician-led maternity units from 1 June 2011 to 31 May 2012 ^11^. The incidence of severe sepsis was 4.7 out of 10,000 maternities, and five women died (1.4%). Genital tract infection (31.0%) and the presence of *E. coli* (21.1%) were the most common causes of sepsis. Women had statistically significantly increased risks of severe sepsis if they were from ethnic minority groups or had co-morbidities. The study concluded that the rapid progression to severe sepsis highlights the importance of following the International Surviving Sepsis Campaign (SSC) guideline of early administration of high-dose intravenous (IV) antibiotics within 1 hour of hospital admission for anyone with suspected sepsis ^12^.

Maternal sepsis can be caused directly by genital tract infections or indirectly by systemic infections such as pneumonia. In the UK, direct infection was the leading cause of maternal death between 2006 and 2008 (1.13 out of 100,000 maternities [26 individuals], having risen from 0.85 out of 100,000 maternities [18 individuals] in the previous triennium) ^13^. The latest triennial report ^14^ of the Confidential Enquiry into Maternal Deaths (2014–2016) revealed a lower incidence of direct infection-related mortality of 0.48 out of 100,000 maternities (11 individuals). This is thought to be due to increased awareness of sepsis among UK obstetricians as a result of the Royal College of Obstetricians and Gynaecologists (RCOG) Green Top Guidelines and the nationwide implementation of the SSC.

However, these figures do not include indirect causes of infection, such as pneumonia or influenza, nor do they account for deaths from major obstetric haemorrhage, secondary to uterine atony or disseminated intravascular coagulation caused by sepsis, which claimed four lives in the UK from 2014 to 2016 ^14^.

## Risk factors for maternal sepsis and septic shock

There are a number of risk factors associated with sepsis and progression to septic shock, which can be categorised as obstetric-related or patient-related risk factors.

### Obstetric-related risk factors

The largest independent obstetric risk factor for postpartum maternal sepsis is operative intervention, and caesarean section (CS) is associated with a 5 to 20% increase in infectious morbidity compared with vaginal birth ^15^. CS after the onset of labour poses the greatest risk, followed by elective CS and then operative vaginal delivery, although antibiotic prophylaxis and sterility are standard practice in the UK ^16^. Other obstetric-related risk factors include cervical cerclage, prolonged rupture of the membranes, a history of pelvic infection, a history of group B streptococcal infection or group A *streptococcus* in close contacts or family members, vaginal discharge, multiple pregnancy, retained products of conception, preterm prelabour rupture of membranes (PPROM) and amniocentesis or other invasive procedures ^10, 13^.

### Patient-related risk factors

According to the UK Obstetric Surveillance System report, patient-related risk factors for maternal sepsis include primiparity, pre-existing medical conditions, ethnic minority status, febrile illness or antibiotic use in the 2 weeks prior to presentation ^11^. Co-morbidities which have an independent association with maternal sepsis include congestive heart failure, chronic liver or renal failure, human immunodeficiency virus infection, systemic lupus erythematosus and diabetes ^10, 13^. The incidence of maternal sepsis is also a prime example of health inequality. There is a strong social gradient associated with maternal sepsis, and the incidence of maternal sepsis is significantly and progressively associated with lower socioeconomic status. In the US, reliance on healthcare through Medicaid is independently associated with development of maternal sepsis ^10, 16^. Furthermore, socioeconomic deprivation is associated with a higher incidence of CS, which in itself is an independent risk factor for developing maternal sepsis ^16^.

## Sources of infection and causative microorganisms

The most common source of maternal infection in the UK is pneumonia, followed by genital tract sepsis. Pneumonia is more common in the intrapartum period, and genital tract sepsis, in association with vaginal birth and obstetric interventions, is more common in the postpartum period ^16^.

### Group A streptococcal genital tract infections

In the UK, the spike in maternal mortality from sepsis during the 2006–2008 triennium was attributed to an increase in group A streptococcal ( *Streptococcus pyogenes*) genital tract infections, which were responsible for 50% of direct maternal deaths ^13^. Group A *streptococcus* is a common cause of bacterial throat infections in children, and all 13 pregnant women who died from group A streptococcal genital tract infections from 2006 to 2008 in the UK either had worked or were working closely with young children. In addition, streptococcal pharyngitis is most common between December and April, which corresponds to the peak timing of maternal deaths.

With its array of surface proteins such as lipoteichoic acid and M proteins that adhere to and allow invasion of epithelial cells, *S. pyogenes* can invade intact epithelium. This facilitates invasive infections such as necrotising fasciitis, genital tract infections, or pneumonia ^17^. Once *S. pyogenes* has invaded the host, it can evade phagocytosis because of the presence of a hyaluronic acid capsule, which allows multiplication within the host ^18^. Following invasion, *S. pyogenes* can cause toxic shock syndrome due to super-antigens such as streptococcal pyrogenic exotoxins, which directly activate T cells without the need for an antigen-presenting cell. This causes massive cytokine production and subsequent multiple organ failure ^19^. The UK Obstetric Surveillance System identified that group A streptococcal genital tract infections were strongly associated with progression to septic shock and were associated with worse outcomes than *E. coli* infections, although *E. coli* is the most common cause of genital tract infections. About 50% of patients with proven group A streptococcal infections progress to septic shock with a greater rapidity in deterioration compared with infections by other organisms ^11^. Since postpartum women are 20 times more likely than non-pregnant women to develop a group A streptococcal infection, awareness of the infection is essential to reduce maternal mortality from sepsis ^20^.

### *Escherichia coli* 

A prospective review of 150,043 pregnancies between 2005 and 2012 identified that *E. coli* was the most common pathogen, accounting for 37% of maternal sepsis cases, and was the predominant pathogen in the antenatal period ^21^. A further analysis highlighted that *E. coli* infection was most common in the third trimester, and 55% of cases were urinary tract infections and 45% of cases were genital tract infections ^22^. In that study, 27% of *E. coli* infections resulted in fetal death, which was due mainly to chorioamnionitis following PPROM, demonstrating the severe impact that bacteraemia has on fetal outcomes ^22^.

### Influenza

Influenza virus infections contribute significantly to the incidence of maternal sepsis, particularly during pandemic years. Influenza symptoms are more severe in pregnancy and result in a four- to five-fold increase in serious illness and the need for hospitalisation ^23^. Influenza infection during pregnancy is most common in the second and third trimesters and in the early postpartum period and is associated with higher rates of preterm birth and poor fetal growth ^24^. The novel H1N1 strain of influenza A resulted in the swine flu pandemic which reached its peak in 2009 and, according to the Centers for Disease Control and Prevention, claimed the lives of 30 women, which was 5% of total deaths from H1N1 in the US in 2009 ^25^. The Mothers and Babies: Reducing Risk through Audits and Confidential Enquiries across the UK (MBRRACE-UK) report from 2009 to 2012, which coincided with the swine flu pandemic, found that 1 in 11 maternal deaths was due to influenza infections in the UK and Ireland ^26^. Accordingly, influenza vaccination is recommended for all pregnant women at any stage of pregnancy, and pregnant women are listed as a high priority for influenza vaccination by the World Health Organization (WHO) ^27^. Uptake of the influenza vaccine by pregnant women has been suboptimal; the percentage of pregnant women who received the vaccine in the UK in 2017/18 was only 47%, which is far below the WHO-recommended uptake of 75% ^28^. Oseltamivir, a neuraminidase inhibitor, is recommended as a first-line therapy for pregnant women infected with influenza and, given within the first 48 hours of infection, can reduce the severity of symptoms and the length of illness ^29^. 

## Diagnosing maternal sepsis

### A move away from systemic inflammatory response syndrome criteria

The international definition of sepsis was changed in 2016 by the Third International Consensus Definitions for Sepsis and Septic Shock (Sepsis-3) Committee ^30^. The definition of sepsis is now “life threatening organ dysfunction caused by a dysregulated host response to infection” ^30^. Further subsets of patients who have “septic shock” include those who require vasopressors to maintain a mean arterial pressure of at least 65 mm Hg and who have a serum lactate level of at least 2 mmol/L. This was a major shift from the previous Sepsis-2 definition, which required patients to have at least two systemic inflammatory response syndrome (SIRS) criteria as well as a confirmed or suspected infection for “sepsis” to be diagnosed. Patients who met these criteria and had organ dysfunction were classed as having “severe sepsis”, and patients who had hypotension which did not respond to fluid resuscitation were classed as having “septic shock”.

The new Sepsis-3 definition has removed the definition of sepsis without organ dysfunction, made the term “severe sepsis” redundant, and has removed the SIRS criteria which were previously used to screen for sepsis. The committee felt that SIRS focussed inappropriately on inflammation rather than organ dysfunction, and the authors concluded that the SIRS criteria, as a screening tool, lacked validity since 1 in 8 patients admitted to intensive care units (ICUs) with infection and new organ dysfunction did not meet the SIRS criteria ^31^. The authors also felt that, according to a retrospective analysis ^32^, SIRS criteria lacked discriminant validity as many patients who did not develop infection or organ dysfunction meet the SIRS criteria.

To match the diagnosis of sepsis with the new definition, the committee decided to use the sequential organ failure assessment (SOFA) scoring system. SOFA assesses the function of multiple organ systems (respiratory, coagulation, liver, cardiovascular, central nervous system, and renal) on a scale from 0 to 4 on the basis of a variety of parameters. It is commonly used in the ICU to predict mortality, where higher scores are associated with poorer outcomes. Organ dysfunction was defined as an acute change in the SOFA score of at least 2 points. This was predictive of a 10% mortality rate in patients who were suspected of having an infection ^33^. The baseline SOFA score was considered to be 0 in patients without pre-existing organ dysfunction. Calculating the SOFA score requires variables such as partial pressure of arterial oxygen (PaO _2_), bilirubin, platelet count, creatinine and urine output, which are impractical during the initial assessment of a patient with suspected sepsis. Accordingly, a quick SOFA (qSOFA) score has been advocated by the Sepsis-3 committee for patients outside the ICU. This score uses three variables that have been demonstrated, through multivariate logistic regression analysis, to predict inpatient mortality ^30^. These variables are tachypnoea (respiratory rate of at least 22 breaths per minute), hypotension (systolic blood pressure of not more than 100 mm Hg) and altered level of consciousness (Glasgow Coma Score scale of not more than 14), and patients having at least two of these features are classed as being at high risk of poor outcomes following sepsis. Outside the ICU, qSOFA, compared with SIRS and SOFA, is a better predictor of mortality. Septic patients with a qSOFA score of at least 2 had a mortality rate of 24% ^34^. These changes are summarised in Table 1.

**Table 1.** A summary of the change in the definitions and approach to patients with suspected sepsis.

	Sepsis	Severe sepsis	Septic shock
Old Sepsis-2 Definitions (2001)	Infection + at least two systemic inflammatory response syndrome (SIRS) criteria SIRS criteria: 1. Tachycardia (heart rate >90 beats per minute) 2. Tachypnoea (respiratory rate >20 breaths per minute) 3. Fever or hypothermia (temperature >38°C or <36°C) 4. Leucocytosis (white cell count >12 g/L), leukopenia (white cell count <4 g/L) or bandemia (>10% immature neutrophils in blood)	Sepsis + evidence of organ dysfunction or tissue hypoperfusion or hypotension	Severe sepsis + refractory hypotension
Current Sepsis-3 Definitions (2016)	Infection + organ dysfunction Organ dysfunction defined as a sequential organ failure assessment (SOFA) score of at least 2 Alternatively, fulfilling at least two of the following quick SOFA criteria correlates with a high risk of mortality (>24%) and should prompt further investigation of organ dysfunction. 1. Hypotension (systolic blood pressure <100 mm Hg) 2. Altered mental status (Glasgow Coma Scale score <15) 3. Tachypnoea (respiratory rate >22 breaths per minute)	Not applicable	Vasopressors required to maintain a mean arterial pressure of at least 65 mm Hg + serum lactate level of at least 2 mmol/L

The new guidelines have removed the term “severe sepsis” and require evidence of organ dysfunction in order for a patient to be classed as being septic.

### The challenges of diagnosing maternal sepsis

The physiological adaptations of pregnancy can make the clinical signs of sepsis more insidious in pregnant women. Pregnancy is associated with a hyperdynamic circulation, and there is a 30 to 50% increase in circulating volume by 28 weeks of gestation. This hyperdynamic circulation can mask cardiovascular signs of sepsis, when, owing to vasodilation, pregnant women experience a drop in systolic and diastolic blood pressure, particularly in the first trimester, and a compensatory sinus tachycardia ^35^. Tachypnoea caused by sepsis can be confused with the physiological tachypnoea in pregnancy caused *inter alia* by elevated progesterone levels. Maternal physiological parameters overlap with current SIRS criteria, so modifications to SIRS criteria are required to identify maternal sepsis ^36^. Apart from temperature, all other components of the SIRS criteria overlap with the physiological parameters of healthy pregnant women during the second and third trimesters and intrapartum. In addition, the qSOFA score includes components that may overlap with maternal physiology. The lack of a rapid screening tool that incorporates physiological changes in pregnancy has been blamed for delays in the diagnosis of maternal sepsis. This was a common and avoidable contributing factor in many of the cases during the spike in deaths from maternal sepsis in the UK between 2006 and 2008 ^7^. In the UK, the RCOG recommends the use of the Modified Early Obstetric Warning System (MEOWS) to detect signs of sepsis and to trigger escalation to senior review of patients with features of concern, as it has been demonstrated to have an 89% sensitivity and 79% specificity in identifying maternal morbidity when validated amongst 676 patients in a UK hospital ^37^. The parameters included in MEOWS are outlined in Table 2. Many other obstetric early-warning systems, such as the Maternal Early Warning Score (MEWS) and the Maternal Early Warning Trigger Tool (MEWT), are available. These tools, particularly the MEWT tool, which is aimed at early identification and treatment of the four commonest causes of maternal morbidity (sepsis, haemorrhage, cardiopulmonary dysfunction, and hypertension), have shown promise. When first introduced, this reduced severe maternal morbidity by 18% ^38^. However, the positive predictive value (PPV) of these tools for sepsis is low. The MEWT has a 7% PPV for sepsis, and six other early warning scores have a sensitivity of between <2 and 15% for sepsis in women with chorioamnionitis. This emphasises that the identification of sepsis cannot be provided by a single tool but requires an individual, holistic approach ^38, 39^.

**Table 2.** The parameters included in the MEOWS chart *.

Parameter	Red trigger	Yellow trigger
Temperature, °C	<35 or >38	35–36
Systolic blood pressure, mm Hg	<90 or >160	150–60 or 90–100
Diastolic blood pressure, mm Hg	>100	90–100
Heart rate, beats per minute	<40 or >120	100–120 or 40–50
Respiratory rate, breaths per minute	<10 or >30	21–30
Oxygen saturation, percentage	<95	N/A
Pain score	N/A	2–3/10
Neurological response	Unresponsive or responsive only to pain	Responsive to voice but not alert
If the patient scores two yellow triggers or one red trigger, a senior anaesthetist AND obstetrician must be called.		

*These parameters have been advocated for use in all obstetric units by the RCOG to identify critical illness in the pregnant population early and to facilitate early intervention and response by senior doctors. This tool was validated for use in 2011 in the UK ^37^. MEOWS, Modified Early Obstetric Warning System; N/A, not applicable; RCOG, Royal College of Obstetricians and Gynaecologists.

## Management of maternal sepsis

### Identification of maternal sepsis

Since 2004, the SSC has published protocols for the initial management of patients with sepsis. The latest 2018 “Hour-1 bundle” consists of five elements of care, which should be initiated within the first hour of the recognition of sepsis ^40^. The elements are lactate measurement, blood cultures prior to antibiotics, administration of broad-spectrum antibiotics, administration of a 30-mL/kg crystalloid fluid bolus in cases of hypotension or high serum lactate levels (hyperlactataemia) of at least 4 mmol/L, and administration of vasopressors to maintain a mean arterial pressure of at least 65 mm Hg. The UK Sepsis Trust has adapted the SSC bundle to include six elements known as the “Sepsis Six”, which also include administration of high-flow oxygen and monitoring of urine output within the first hour of recognition of sepsis. Simplified pathways, such as the Sepsis Six, have been shown to increase delivery of all elements of the SSC bundle and to reduce mortality by up to 50% ^41^. Measurement of serum lactate is advocated in sepsis, as hyperlactataemia is a marker for anaerobic metabolism subsequent to tissue hypoperfusion, although other factors such as mitochondrial dysfunction, microcirculatory failure, reduced oxygen extraction, increased glycolytic flux due to an endogenous catecholamine surge during sepsis, and hepato-renal dysfunction (70% of lactate is eliminated by the liver), resulting in decreased lactate elimination, have been implicated ^42^. Elevated lactate has been positively associated with the need for ICU admission in obstetric patients, and every 1-mmol/L increase in lactate is associated with a 2.34-fold increased risk in the need for ICU admission ^43^. Accordingly, lactate may permit early identification of pregnant women with sepsis, who need immediate critical care.

### Antibiotics in maternal sepsis

The use of early, appropriate antibiotics is crucial in the management of maternal sepsis. Accordingly, they play an important role in the SSC guidelines. The importance of early antibiotics is highlighted by a retrospective analysis of 2731 ICU admissions for septic shock, which showed a 7.6% decrease in survival for every hour of delay in antibiotic administration after the onset of hypotension ^44^. Early involvement of infectious disease specialists is also recommended when making decisions about antimicrobial therapy ^13^.

Initial antibiotics administered in sepsis should be broad-spectrum, administered within one hour of suspected sepsis, after blood for culture has been taken. Genital tract infections are often polymicrobial, and group A *streptococcus* and *E. coli* are commonly associated with severe infections, hence the need for empirical broad-spectrum antibiotics that cover Gram-positive, Gram-negative and anaerobic organisms before culture results are available. The exotoxins produced by group A *streptococcus* can cause rapid deterioration and streptococcal toxic shock syndrome. Clindamycin has been shown to inhibit exotoxin production by group A *streptococcus* and should be administered with broad-spectrum antibiotics to improve clinical outcome ^45^. Administration of IV immunoglobulin to neutralise exotoxins may improve outcomes from toxic shock syndrome and is recommended by the RCOG ^13, 46^. Prophylactic antibiotics prior to operative obstetric intervention should be considered mandatory, and prophylaxis with IV azithromycin in addition to IV cefazolin, compared with the use of IV cefazolin alone, results in a significant reduction in the incidence of postoperative infections, endometritis and wound infection ^47^.

### Ventilation strategies in the management of pregnant women with severe sepsis

Ventilation strategies for septic pregnant women may have to be adapted from the general population with sepsis. The maternal arterial PaO _2_ should be maintained at more than 70 mm Hg and partial pressure of carbon dioxide (PaCO _2_) at less than 60 to 70 mm Hg to ensure fetal oxygenation and placental perfusion ^48^. Acute respiratory distress syndrome (ARDS) has a mortality rate of 23% in the antepartum period and 50% in the postpartum period ^48^. Prone ventilation in pregnancy is associated with significant improvements in oxygenation of pregnant women with severe ARDS ^49, 50^. The PROSEVA (Proning Severe ARDS Patients) trial demonstrated a reduced mortality in severe ARDS with prone ventilation, and the 2016 SSC guidelines recommend that prone ventilation should be administered to adults with ARDS of septic origin with a PaO _2_/FiO _2_ (PaO _2_/fraction of inspired oxygen) ratio of less than 150. Accordingly, prone ventilation may become increasingly used in pregnant women with sepsis ^51^. In addition, the SSC strongly recommends low tidal volume ventilation in ARDS of 6 mL/kg and an upper limit plateau pressure of 30 cm H _2_O on the basis of evidence from several clinical trials that have demonstrated benefit from lung-protective ventilation strategies ^52^.

### Extracorporeal membrane oxygenation

As a treatment for respiratory failure in patients in the ICU, extracorporeal membrane oxygenation (ECMO) has been used increasingly during pregnancy, particularly during the H1N1 epidemic of 2009. There were fears of potential fetal harm and bleeding with ECMO, but outcomes were comparable to those of the non-pregnant population. The rates of maternal and fetal survival were 80% and 70% respectively, and there was no significant increase in haemorrhage ^53^. The dangers of refractory hypoxia on maternal and fetal outcome, combined with higher mortality rates with ARDS in pregnancy, make ECMO an increasingly viable rescue therapy. Delivery of the fetus may be necessary in cases of persistent maternal hypoxia since this may improve maternal ventilation and oxygenation through improved lung compliance and less splinting of the diaphragm. However, case series have expressed caution with this assumption ^54^.

## Case studies

We present summaries of two case studies from 2018. Both cases were reviewed by one of the co-authors (RFL) to produce medicolegal reports, one of which was requested by a hospital in preparation for a Coroner’s Court Enquiry.

### Septic shock following preterm prelabour rupture of membranes

***1.*** *A multiparous, 38-year-old woman at 15 ^+3^ gestation presented to Accident and Emergency (A&E) complaining of pink vaginal discharge and pelvic pressure. A speculum examination confirmed the diagnosis of PPROM. Oral erythromycin was administered, and initial vital signs were within the normal range. Blood results revealed a leucocytosis with a white cell count (WCC) of 13.1, an elevated C-reactive protein (CRP) of 25 mg/L and anaemia (haemoglobin of 107 g/L). Ultrasound revealed a live fetus and oligohydramnios. The patient was admitted to hospital for observation*.

***2.*** *On day 3 post-admission, the patient agreed to a medical termination of pregnancy but deteriorated and complained of rigors, nausea, lower abdominal pain, frontal headaches and generalised pain. She was tachypnoeic, and her lowest systolic and diastolic blood pressures recorded during this period were 89 and 53 mm Hg respectively. She was pyrexial (38.9 °C), and the Sepsis Six protocol was commenced. Bloods, high vaginal swab and a midstream urine were sent off to microbiology, and a fluid bolus and IV co-amoxiclav were administered. The blood tests revealed a rise in CRP to 47 mg/mL, a WCC of 7.3, an elevated serum lactate of 2.2 mmol/L and a normal pH of 7.4. The lower abdomen was tender, and a vaginal speculum examination revealed that the cervical os was open and that fetal parts were visible, confirming that a spontaneous abortion was in progress. The obstetric team decided to continue with conservative management with a plan to administer the uterotonic misoprostol in 4 to 6 hours’ time if required*.

***3.*** *Overnight, the patient complained of worsening abdominal pain. Her heart rate was 96 and her blood pressure was 87/40 mm Hg. The cervix was dilated 1 cm on vaginal examination, and there was no progression of the miscarriage. Various medical and surgical opinions were sought*.

*At 3 a.m. on day 4 of admission, the patient continued to deteriorate and had signs of multi-organ failure. Her latest venous blood gas showed a profound metabolic acidosis with a pH of 7.13, a base excess of – 16.3 mEq/L and a serum lactate level of 12 mmol/L. The patient was hypoxic in room air, required 3 L of oxygen via nasal cannulae to reach an oxygen saturation of 96%, and had a respiratory rate of 32. Her blood pressure at this stage was 80/47 mm Hg, and her heart rate was 108. A positive fluid balance of 2215 mL was recorded on the fluid chart. Misoprostol was prescribed, and an ICU referral was made.*

***4.*** *By 5:30 a.m., the patient was bleeding from her urinary catheter site while in the ICU, and her platelet count was 17/μL, indicating disseminated intravascular coagulation (DIC). Her blood pressure was now 79/57 mm Hg*.

***5.*** *At 6:30 a.m., for advice on antibiotic therapy, a decision was made to contact the microbiologist, who recommended changing from IV co-amoxiclav to IV tazocin. In addition, blood transfusions were commenced to address the DIC, and over the next 3 hours, 5 units of fresh frozen plasma, 2 units of cryoprecipitate and 2 units of platelets were transfused. An evacuation of retained products of conception (ERPC) was scheduled for when the patient had been stabilised*.

***6.*** *At 8:09 a.m., the ERPC was commenced. The fetus was partially in the vagina and was removed manually, the products of conception were malodorous and the uterus was evacuated by suction curette. The patient returned to the ICU intubated and ventilated, IV gentamycin was added to her antimicrobial therapy, and haemodialysis was initiated. She remained hypotensive and peripherally shut down for the remainder of the day in spite of inotropic support*.

***7.*** *On day 5 of admission, the blood cultures reported a growth of E. coli. In the afternoon, the patient became bradycardic and then asystolic and she could not be resuscitated in spite of of 20 cycles of cardiopulmonary resuscitation.*

***8.*** *This patient had obstetric risk factors for maternal sepsis, including PPROM and retained products of conception, as well as patient risk factors such as ethnic minority status. The causative organism for her septic shock was E. coli, the most common cause of maternal genital tract infections in the UK. The first sign of sepsis was in the afternoon of day 3 post-admission, when the patient would have scored 2 on the qSOFA scale because of hypotension and tachypnoea, which signifies a high mortality risk. In addition, she had an elevated serum lactate of 2.2 mmol/L, which would indicate sepsis and the need for escalation to intensive care. The source of infection was clearly uterine, and it was deemed that the delay in source control (ERPC) that was performed 19 hours after the initial deterioration, and the request for opinions from other medical and surgical specialities, resulted in a missed opportunity to save the patient’s life. There was also a delay in seeking expert advice from an infectious disease specialist as advocated by the RCOG, and the switch to IV tazocin and gentamicin occurred only after DIC had been diagnosed on day 4 post-admission.*

### Septic shock following postpartum pyelonephritis

**1.** *A 28-year-old, primigravida of South Asian ethnicity with type 2 diabetes had an elective CS at 37 weeks’ gestation for a persistent breech presentation. During pregnancy, the patient was treated for three confirmed E. coli urinary tract infections. She was discharged from hospital on day 2 postoperatively.*

**2.** *On the morning of day 4, the patient developed rigors. Her husband called the hospital’s midwifery team but was unable to get through. In the evening, the husband spoke to a midwife by phone and was asked to record his wife’s temperature, which was normal. The husband expressed concern that this may have been due to the regular use of paracetamol but was advised by the midwife not to bring the patient into hospital and that the symptoms were probably pain-related.*

**3.** *On the morning of day 5, the patient felt nauseous and had two episodes of vomiting and rigors. A midwife arrived at midday for a routine appointment and checked the wound site but was not carrying a thermometer and therefore could not take the patient’s temperature. At the husband’s request, the midwife booked a general practitioner (GP) appointment for that afternoon.*

**4.** *When the patient was assessed by the GP at 4:45 p.m., she was found to be tachycardic, hypotensive, tachypnoeic and pyrexial. The GP arranged for an ambulance to make an emergency transfer to A&E, where the patient arrived at 6:38 p.m. In A&E, she was found to be hypoxic on room air and had an elevated lactate. The Sepsis Six protocol was immediately instituted, and on the advice of a microbiologist, IV tazocin was administered. The patient continued to deteriorate, and she was admitted to the ICU, where she was intubated and placed on inotropic support. Despite this, she suffered from seven pulseless electrical activity (PEA) cardiac arrests and was pronounced dead at 3 a.m. on day 6 postnatally. Post-mortem results demonstrated* E. coli *isolation from the abdominal wound, the uterine incision, the bladder, the right lung and blood.*

***5.*** *The patient had multiple obstetric and patient risk factors for sepsis, including nulliparity, diabetes, ethnic minority status, antibiotic use within the preceding 2 weeks prior to hospital admission, and CS. The causative organism for her septic shock was E. coli. This case highlights the rapid progression to septic shock, and the patient died just over 24 hours after initial symptoms of infection. Accordingly, prompt identification and aggressive intervention are crucial in the management of sepsis. The patient faced a delay of 14 hours from symptoms of infection to diagnosis of sepsis by the GP. It was deemed that the assessments made by midwives prior to admission to hospital were inadequate and resulted in a delay in identification and management of sepsis with IV antibiotics. The patient’s risk factors, combined with her infective symptoms, should have prompted the suspicion of sepsis, which would possibly have saved her life.*

## Conclusions

Maternal sepsis remains a significant cause of morbidity and mortality in pregnancy. The terminology of sepsis has recently changed, and it is important from both a clinical and research viewpoint to remain up to date and understand this change. Further research into risk factors for maternal sepsis is required to reduce the incidence and to facilitate early identification and treatments that were previously considered not feasible in pregnant women. Interventions such as ECMO and prone ventilation have gained increasing support and require larger studies to assess their role in the management of maternal sepsis.

## Abbreviations

A&E, Accident and Emergency; ARDS, acute respiratory distress syndrome; CRP, C-reactive protein; CS, caesarean section; DIC, disseminated intravascular coagulation; ECMO, extracorporeal membrane oxygenation; ERPC, evacuation of retained products of conception; GP, general practitioner; H1N1, haemagglutinin 1 neuraminidase 1; ICU, intensive care unit; IV, intravenous; MEOWS, Modified Early Obstetric Warning System; MEWT, Maternal Early Warning Trigger Tool; PaO _2_, partial pressure of arterial oxygen; PPROM, preterm prelabour rupture of membranes; PPV, positive predictive value; qSOFA, quick sequential organ failure assessment; RCOG, Royal College of Obstetricians and Gynaecologists; SIRS, systemic inflammatory response syndrome; SOFA, sequential organ failure assessment; SSC, Surviving Sepsis Campaign; WCC, white cell count; WHO, World Health Organization
